# Omission of perioperative morphine reduces postoperative pain in proctological interventions: a single-center analysis

**DOI:** 10.1007/s13304-023-01640-2

**Published:** 2023-09-05

**Authors:** Fabian Haak, Fabio Nocera, Lorena Merlo, Belma Dursunoglu, Silvio Däster, Fiorenzo V. Angehrn, Daniel C. Steinemann

**Affiliations:** 1 Clarunis, Department of Visceral Surgery, University Center for Gastrointestinal and Liver Diseases, St. Clara Hospital and University Hospital Basel, Postfach, 4002 Basel, Switzerland; 2 Medical Faculty, University of Basel, Basel, Switzerland

**Keywords:** Proctology, Pain, Anesthesia and analgesia, Morphine

## Abstract

There is an increase in outpatient procedures and this trend will continue in the future. For hemorrhoidectomy, it is the standard of treatment in many health care systems. Perioperative management including adequate pain control is of paramount importance to ensure successful ambulatory surgery. This study investigates the role and effect of morphine compared to short-acting opiates applied before, during, or after proctological interventions and with focus on hemorrhoidectomy. A retrospective analysis of a prospective database was conducted comparing two populations. The control cohort received morphine (Yes-Mô) intra- and postoperatively, while the intervention group did not receive morphine (No-Mô) between January 2018 and January 2020. Both cohorts were balanced by propensity score matching. The outcomes were postoperative pain measured by numeric ratings scale (NRS) one hour postoperatively, pain 24 h postoperatively, success rate of outpatient management, and complication rate including postoperative nausea and vomiting as well as urinary retention. The intervention population comprised 54 patients and the control group contained 79 patients. One hour after surgery, patients in No-Mô reported lower NRS (1.44 ± 1.41) compared to Yes-Mô (2.48 ± 2.30) (*p* = 0.029). However, there was no difference in NRS 24 h postoperatively (No-Mô: 1.61 ± 1.41 vs Yes-Mô: 1.63 ± 1.72; *p* = 0.738). 100% of No-Mô was managed as outpatients while only 50% of Yes-Mô was dismissed on the day of the operation (*p* = < 0.001). There was no difference in postoperative complications (including postoperative nausea and vomiting (PONV) and urinary retention) between the two groups (PONV No-Mô 7.4% vs Yes-Mô 5.6%, *p* = 1.0 and urinary retention No-Mô 3.7% vs Yes-Mô 7.4%, *p* = 0.679). No-Mô received an oral morphine equivalent of 227.25 ± 140.35 mg intraoperatively and 11.02 ± 18.02 mg postoperatively. Yes-Mô received 263.17 ± 153.60 mg intraoperatively and 15.97 ± 14.17 mg postoperatively. The difference in received morphine equivalent between the groups was not significant after matching for the intraoperative (*p* = 0.212) and postoperative (*p* = 0.119) received equivalent. Omission of perioperative morphine is a viable but yet not understood method for reducing postoperative pain. Omission of morphine leads to a lower use of total morphine equivalent to attain satisfactory analgesia. The reduction of the overall opiate load and using opiates with a very short half-life potentially leads to a reduction of side effects like sedation. This in turn promotes discharge of the patient on the day of surgery. Omission of morphine is safe and does not increase postoperative complications.

## Introduction

Outpatient surgery is becoming increasingly important. Due to improvements of perioperative care and an increased financial pressure on health care systems discharge at the day of surgery is becoming an important goal [1]. Proctological interventions such as hemorrhoidectomy already represent this trend at the current time [2, 3]. As early as 2003, the American Society of Colorectal Surgery stated that 90% of anorectal surgery patients could be managed on an outpatient basis [4]. In Switzerland, in 2019, laws went into effect making it mandatory to perform hemorrhoidectomies in an outpatient setting in order to receive reimbursement [5]. Only patients fulfilling exception criteria (e.g., Heart Insufficiency New York Heart Association > Grade II, chronic obstructive pulmonary disease > Grade II, therapeutic anticoagulation, chronic kidney disease > Grade III) are exempted from this regulation [6]. This has been accepted and pursued by the majority of the involved practitioners [7].

A shortened hospital stay reduces postoperative complications and morbidity [8, 9]. Therefore, a short hospital stay has been targeted in the last decade not only for financial reasons. Although outpatient surgery is common practice, its role in this context especially if there might even be a further decreased morbidity compared to short hospital stay management has yet to be investigated by prospective, randomized comparative studies [10]. The surgical technique for the treatment of hemorrhoids is of importance but selected procedures (Milligan and Morgan, Ferguson procedure, stapled hemorrhoidopexy, Doppler-guided hemorrhoidal artery ligation) are comparable in regard of successful outpatient management [10].

Adequate postoperative pain management is eminent for successful outpatient surgery. Pain exacerbation may hamper discharge as patients trust in self-management is compromised. As will be mentioned later, there are several contributing factors to postoperative pain. But the choice of procedure does not have an impact [11].

In this context, the choice of analgesia is of the upmost importance. Morphine and fentanyl or derivates are commonly used. Morphine is naturally found in the poppy plant (*Papaver somniferum*). It is mainly an agonist of the μ–δ-opioid receptor in the central nervous system. [12] Binding and activation of this receptor is associated with analgesia, sedation and respiratory depression. Plasma peaks are reached 20 min after intravenous injection and 30min after oral administration [13]. After metabolization by the liver morphine is renally eliminated with a half-life of approximately 120 min [14]. Possible side effects of morphine include decreased respiration, vomiting or nausea, low blood pressure, and obstipation [15]. Morphine is the standard opiate to which other opioids are compared [14]. One such other opioid is remifentanyl (Ultiva™, Glaxo Welcome, Inc., Research Triangle Park, NC). It has agonist activity at the μ-receptor and is comparable to fentanyl in potency, therefore being approximately one hundred times more potent than morphine when applied intravenously [16]. It has a rapid onset of action (1 min) and offset of action (3–10 min). The most commonly occurring side effects of remifentanyl relate to its agonist receptor properties and are, therefore, comparable with morphine [16].

This study investigates the effect of perioperative analgesia and the specific effect of morphine on postoperative pain compared to a pain management including short-acting opiates instead. As described above, this is a central determinant of successful outpatient management.

## Methods

Two populations of patients were analyzed. Data were collected prospectively using a standardized form by a dedicated nurse.

The intervention population No-Mô did not receive morphine during surgery nor in the perioperative course. Patients belonging to this group were treated between February 2020 and March 2021. The control population Yes-Mô received morphine either intra- and/or perioperatively. Patients in Yes-Mô were treated between January 2018 and January 2020. This analysis therefore investigates a clear perioperative regimen change at a specific timepoint (February 2020). A detailed description of perioperative pain management is provided in Table 1.

**Table 1:**
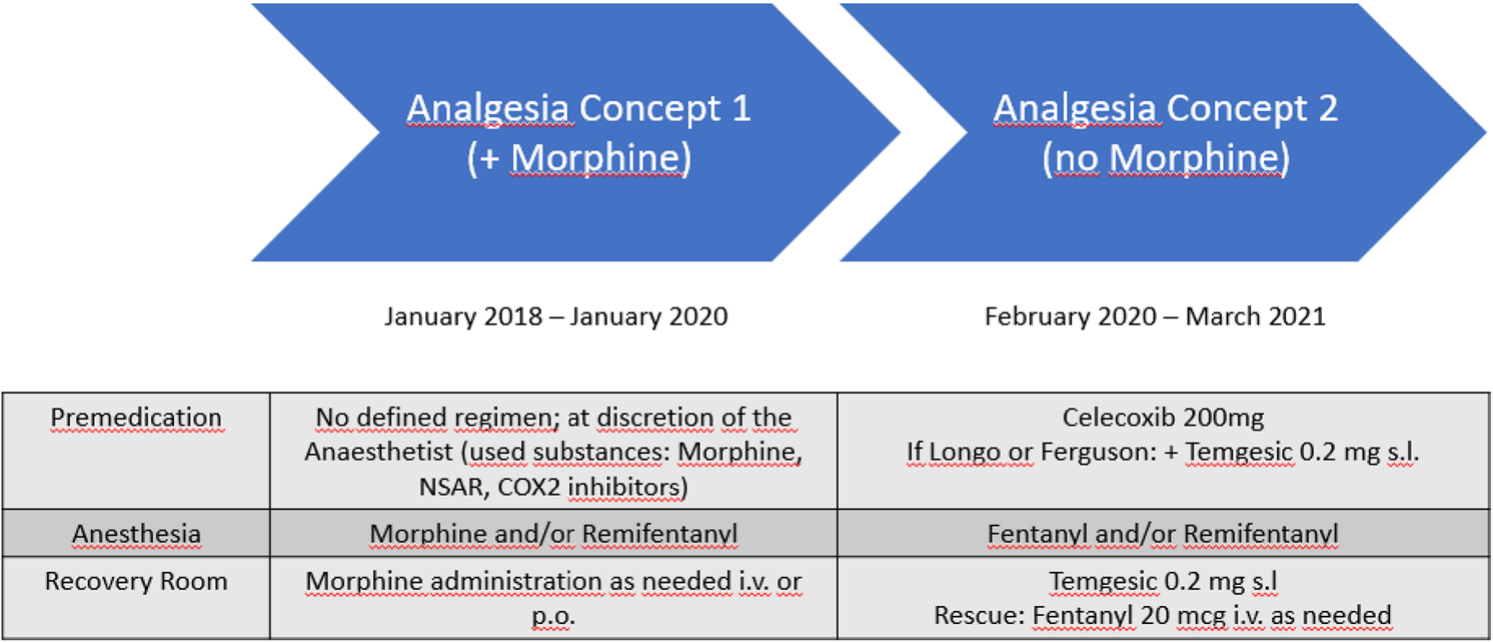
Analgesia

The following surgical procedures are included in this study: stapler hemorrhoidectomy, closed hemorrhoidectomy, open hemorrhoidectomy, and excisional skin tag removal. Only procedures performed under general anesthesia are included in this study. A perianal block using ropivacaine was applied by choice of the surgeon in some patients. Concerning technique and reason for perianal block we refer to previously published results by our research group [17].

To compensate for differences between the groups in key co-variables (diagnosis and percentage of perianal block applied), a propensity score analysis was performed to match the populations. Difference-in-means of covariates of the two groups was evaluated. The propensity score was estimated by running a logit model with the outcome variable being binary indicating the treatment group. Using this model, a propensity score was calculated for each patient. The used matching algorithm finds pairs of observations that have very similar propensity scores, but differ in their treatment status [18].

Endpoints of this study are pain measured one hour and 24 h after surgery, successful outpatient management, and rate of complications. Severity of complications were defined by Clavien–Dindo classification [19]. Pain was quantified using a numeric rating scale (NRS) collected by a dedicated nurse face to face (one hour after surgery) and by telephone (24 h after surgery), respectively [20]. Outpatient management consists of a dedicated physician providing a follow-up by telephone using standardized protocols and questionnaires [21]. The telephone follow-up was conducted by an independent physician not associated with the performed surgery. Outpatient management was considered successful if the patient was able to be dismissed before closing hour of the outpatient clinic. Urinary retention and postoperative nausea and vomiting (PONV) were considered as postoperative complications.

Descriptive statistics were used to analyze the quantitative data. An unpaired or paired *t*-test was used to compare continuous data. Fisher’s exact test and Mann–Whitney test was used for analysis of categorical variables. Propensity score was estimated running a logit model. Pairs were identified that have nearest propensity scores but differ in treatment status. All analyses were done using R (R Core Team) and matching was performed using package MatchIt by Ho et al.[18]

## Results

### Pre matching

The intervention population comprised 54 patients and the control group contained 79 patients. There was no difference in age between the two groups (Mean No-Mô 50.7 ± 12.9 vs 52.1 ± 13.5 years in Yes-Mô, *p* = 0.550). The distribution between sex in both groups was similar (63% male in No-Mô vs 59.5% in Yes-Mô, *p* = 0.689). The American Society of Anesthesiologists (ASA) classification was the same in both groups (Median ASA 2 (IQR 2–2) in No-Mô vs 2 (IQR = 2–2) in Yes-Mô, *p* = 0.760).

There was a significant difference in diagnosis between the two groups (*p* = 0.020). In Yes-Mô, more patients had hemorrhoids grade II (15 vs 4). Yes-Mô had a larger amount of hemorrhoids grade III compared to No-Mô. In contrast, No-Mô had five patients with hemorrhoids grade IV compared to none in Yes-Mô. The patients defined as having other diagnosis mostly had perianal skin tags removed (Table 2).

**Table 2:** Group characteristics

	Unmatched			Matched		
	No-Mô *N* = 54	Yes-Mô *N* = 79	*p* value	No-Mô *N* = 54	Yes-Mô *N* = 54	*p* value
Mean age in years (SD)	50.7 (12.9)	52.1 (13.5)	0.550	50.7 (12.9)	51.1 (12.9)	0.895
Sex male in %	63.0%	59.5%	0.689	63%	56%	0.438
Median ASA (IQR)	2 (2–2)	2 (2–2)	0.760	2 (2–2)	2 (2–2)	0.853
ASA 1	13	14		13	8	
ASA 2	34	56		34	42	
ASA 3	7	9		7	4	
Diagnosis			0.020			0.813
Hemorrhoid degree I	1	7		1	1	
Hemorrhoid degree II	4	15		4	8	
Hemorrhoid degree III	21	32		21	20	
Hemorrhoid degree IV	5	0		5	0	
Hemorrhoid degree undefined	2	3		2	3	
Other^a^	21	22		21	22	
Mean Morphine equivalent received (oral morphine) in mg (SD)						
Intraoperatively	227.25 (± 140.35)	296 (± 160.32)	0.010	227.25 (± 140.35)	263.17 (± 153.60)	0.212
Postoperatively	11.02 (± 18.02)	22.39 (± 18.91)	0.001	11.02 (± 18.02)	15.97 (± 14.17)	0.119
Perianal block received in %^b^	70.4%	51.9%	0.031	70.4%	64.8%	0.542

^a^Mainly removal of anal skin tags, mucosal prolapse, polyps, hypertrophic anal papillae

^b^Ropivacaine 40 ml (10 ml per quadrant)

Next to a significant difference in diagnosis between the two groups, there was also a difference in rate of applied perianal blocks. A perianal block was applied in 70.4% of No-Mô compared to 51.9% in Yes-Mô (*p* = 0.031) (Table 2).

There was also a significant difference between the amount of morphine equivalent administered intraoperatively and postoperatively between the two groups. No-Mô received less morphine equivalent compared to Yes-Mô (Intraoperatively: No-Mô 227.25 (SD ± 140.35) vs Yes-Mô 296 mg (SD ± 160.32); *p* = 0.010 and postoperatively No-Mô 11.02 mg (SD ± 18.02) vs Yes-Mô 22.39 mg (SD ± 18.91); *p* = 0.001) (Table 3).

**Table 3:** Outcomes

	Unmatched			Matched		
	No-Mô *N* = 54	Yes-Mô *N* = 79	*p* value	No-Mô *N* = 54	Yes-Mô *N* = 54	*p* value
NRS 1 h postoperative Mean (SD)	1.44 (1.41)	2.49 (2.42)	0.032	1.44 (1.41)	2.48 (2.30)	0.029
NRS 24 h postoperative Mean (SD)	1.61 (1.41)	1.62 (1.62)	0.838	1.61 (1.41)	1.63 (1.72)	0.738
Successful outpatient management in %	100%	48.1%	< 0.001	100%	50%	< 0.001
Clavien–Dindo grade I	13	22	0.691	13	16	0.665
Urinary retention	2	6	0.472	2	5	0.679
Postoperative nausea and vomiting (PONV)	4	3	0.441	4	2	1
Clavien–Dindo grade IIIb	0	1	1	0	1	1

*Endpoints pre matching* There was a significant difference between the NRS score one hour postoperatively between the two groups (No-Mô 1.44 (SD ± 1.41) vs Yes-Mô 2.49 (SD ± 2.42), *p* value: 0.032). In contrast, there was no difference in NRS score 24 h postoperatively (No-Mô 1.61 (SD ± 1.41) vs Yes-Mô 1.62 (SD ± 1.62), *p* = 0.838). Successful outpatient management was achieved for all No-Mô cases (100%). In comparison, only 48.1% of the patients in Yes-Mô were dismissed from hospital on the day of the surgery (*p* value < 0.001). There was a non-significant difference in complication rates: Clavien–Dindo grade I complications occurred in 13 patients (24.1%) in No-Mô compared to 22 patients (27.8%) in Yes-Mô (*p* = 0.691). Two cases of urinary retention and 4 cases of PONV are included in 13 Grade I Complications in No-Mô. The 22 Grade I complications in Yes-Mô encompassed 6 cases of urinary retention and 3 cases of PONV. There were no higher complication grades recorded in No-Mô. One patient had to be re-operated due to postoperative bleeding (Clavien–Dindo grade IIIb) in Yes-Mô (Table 3).

### Post matching

Propensity score matching led to two groups of 54 patients. Matching led to a successful group alignment for the covariates diagnosis and perianal block. There was no difference between diagnosis (*p* = 0.813) and perianal block application (No-Mô 70.4% vs Yes-Mô 64.8%, *p* = 0.542). Additionally, there was now no significant difference in received morphine equivalent between the groups (Intraoperatively No-Mô 227.25 mg (SD ± 140.35) vs Yes-Mô 263.17 (SD ± 153.60); *p* = 0.212 and postoperatively No-Mô 11.02 (SD ± 18.02) vs Yes-Mô 15.97 (SD ± 14.17); *p* = 0.119) (Table 3).

*Endpoints post matching* The difference in NRS one hour postoperatively remained significant after matching (No-Mô NRS 1.44 (SD ± 1.41) vs Yes-Mô NRS 2.48 (SD ± 2.30), *p* = 0.029). The NRS score 24 h postoperatively was similar (No-Mô NRS 1.61 (SD ± 1.41) vs Yes-Mô NRS 1.63 (SD ± 1.72), *p* = 0.738). After matching, 100% of the intervention group (No-Mô) were successfully managed on an outpatient basis as compared to 50% of the control group (Yes-Mô; *p* < 0.001). Reasons for failure of outpatient management were urinary retention and pain perceived as not manageable outside of the hospital. The mean NRS score in patients which had to be admitted to the hospital was 2.58 (SD ± 2.44). Post matching, there were 16 grade I complications in Yes-Mô (*p* = 0.665). These encompassed 5 cases of urinary retention and 2 cases of PONV. There were 13 grade I complications in No-Mô encompassing 2 cases of urinary retention and 4 cases of PONV (Table 3).

## Discussion

The current study shows a reduction in perceived pain shortly after surgery in the cohort which did not receive morphine (No-Mô). The proportion of patients who could be successfully managed in an outpatient setting was significantly higher in patients that did not receive morphine. Simultaneously, there was no difference in complication rate. This remains true after controlling for differences in the investigation groups.

The reason for improved pain management without morphine remains unclear. Hyperalgesia is a known side effect of opioids [22]. It is particularly investigated for very-short-acting opioids such as remifentanil and can be dealt with using appropriate strategies [23]. Remifentanil should be combined with other not very-short-acting opioids such as fentanyl. The phenomenon occurs regularly after chronic usage. Furthermore, morphine in particular is not known as a prominent perpetrator in this context. The pharmacological differences between morphine and the other opioids and especially the different interaction with receptors are potentially an explanation for our findings [24]. As such, early investigations have shown that a free 3-hydroxl group is essential for activity and reactions [25].

Identifying morphine-specific actions that contribute to higher perceived pain becomes even more complex when we acknowledge that multilevel interactions exist. Pain perception is determined by genetic factors [26, 27].

There are reports of muscle dysfunction after morphine application at the esophagus, large bowel and sphincter of Oddi [28–30]. Even though muscle dysfunction has not been described for the pelvic floor, following evaluation of feedback charts from our ambulatory surgical program we believe that relief of pelvic floor muscle spasm could be an explanation for reduced pain when morphine is omitted [28–32]. Looking at postoperative morbidity, we did not find a difference in urinary retention. This falls in line with findings from other research groups that could not identify morphine as a propagator of urinary retention [33].

Independent from the question why omission of morphine leads to less postoperative pain, its use for clinical application is clear. Our data have shown that omission of morphine leads to a lower use of total morphine equivalent. The reduction of the overall opiate load and using opiates with a very short half-life potentially leads to a reduction of side effects like sedation. This in turn promotes discharge of the patient on the day of surgery. Additionally, in the context of ambulatory surgery, reducing postoperative pain is crucial for successful management. With our intervention, this is achieved without increasing complications proving it to be a safe intervention.

This study has limitations. It is a retrospective analysis, and therefore selection bias is a concern. The two compared groups were treated over two different periods distinguished by specific analgesia regimen changes. It is also important to note that with introduction of the new anesthesia management, the preoperative scheme was also adapted. Additional analgesia in the form of buprenorphine and celecoxib was applied systematically. This provides additional analgesia and could have an influence on postoperative pain perception.

## Conclusion

Omission of perioperative morphine reduces postoperative pain for proctological interventions without increasing morbidity. Instead of morphine a combination of short-acting and very-short-acting opioids promise better results in the context of outpatient surgery.

We propose a standard protocol for the perioperative management of patients receiving proctological interventions. The protocol consists of morphine-free management in addition to applying a perianal block [17] and standardized premedication.

## Data Availability

The data that support the findings of this study are available from the corresponding author upon reasonable request.
